# Graphene with outstanding anti-irradiation capacity as multialkylated cyclopentanes additive toward space application

**DOI:** 10.1038/srep12734

**Published:** 2015-07-30

**Authors:** Xiaoqiang Fan, Liping Wang

**Affiliations:** 1 State Key Laboratory of Solid Lubrication, Lanzhou Institute of Chemical Physics, Chinese Academy of Sciences, Lanzhou, 730000, P.R. China; 2 University of Chinese Academy of Sciences, Beijing 100039, P.R. China; 3 Key Laboratory of Marine Materials and Related Technologies, Zhejiang Key Laboratory of Marine Materials and Protective Technologies, Ningbo Institute of Materials Technology and Engineering, Chinese Academy of Sciences, Ningbo 315201, China

## Abstract

Multialkylated cyclopentanes (MACs), a class of synthetic hydrocarbon fluid have attracted intensive interest as possible space lubricants due to a series of unique physical and chemical properties. Here, we used graphene with high mechanical strength and chemical inertness as lubricant additive to explore its potential for space application. The effects of space irradiation on graphene and the tribological properties of graphene as lubricant additive were firstly investigated in detail under simulated space environment composed of high vacuum, high/low temperature and irradiation. Results demonstrate that graphene not only possesses outstanding anti–irradiation capacity but also significantly improves the space performance and tribological properties of MACs, which depends on the excellent physicochemical properties and high load-carrying ability of graphene as well as more effective separation of the sliding surfaces.

The success of spacecraft missions depends on the accuracy and reliability of various drive mechanisms that in turn are dependent on the good lubrication with low friction and low wear, low mechanical noise and long-term durability. Hence lubricants play a crucial role in controlling friction and wear and ensuring the performance of these mechanisms. Multialkylated cyclopentanes (MACs) consisted of the di– and tri–substituted (2–octylodecyl) cyclopentane have been focused on the potential application for actual space drive mechanisms and microelectromechanical systems (MEMS) due to the good chemical inertness, excellent viscosity properties, low volatility, low pour points and high thermal stability^1^,^2^,^3^. Extensive researches have demonstrated that MACs possess excellent lubrication properties under atmospheric environment on the earth’s surface^4^, whereas the number of experimental reports on the space lubricating performance is limited because the space environment differs greatly from atmospheric environment, which includes high vacuum (HV), high/low temperature (HT/LT), atomic oxygen (AO), ultraviolet irradiation (UV), proton (PR) and electron–beam irradiation (EL), the absence of a gravitational field, and so on^5^,^6^. Although space tribology has been taken into account and been explored for many years, the reliability and life of mechanical components are still not adequate to meet the ever–increasing performance requirements. With the continuous development of machine elements, tribology is now becoming a major roadblock for the human exploration of the universe^7^,^8^. Take into consideration the properties of MACs, it is a relatively ideal choice for space lubrication^9^. However, in our previous study, we found that the structure of MACs could be seriously damaged by high–energy particles which results in poor lubrication for the contact of steel/steel^10^. Toward fulfilling the promised performance requirements of space vehicles, such as higher reliability and accuracy, longer service life, it is essential to use suitable lubricant additive for greatly improving the friction reducing and antiwear performance.

Carbon nanomaterials are being considered for fundamental research and industrial application. Graphene has attracted tremendous attention for its unique physical and chemical properties including outstanding thermal conductivity (~5000 Wm^−1^K^−1^), extraordinary carrier mobility (up to 27000 cm^2^ V^−1^s^−1^), large theoretical specific surface area (2630 m^2^ g^−1^) and excellent mechanical properties (Young’s modulus of around 1100GPa, fracture strength of 125GPa)^11^,^12^,^13^,^14^. These properties hold great promise for widespread applications in biological sensors, nano–composite materials, optical and electronic devices, and lubrication for MEMS/NEMS^15^,^16^,^17^,^18^,^19^,^20^, whereas its potential relevant to the aerospace industry has been not systematically investigated. Graphene used in space will be subject to the harsh space irradiation environment including high–energy AO, PR, EL, heavier ions and transient particles^21^,^22^,^23^. Although it is not feasible to conduct an exhaustive study of the effects of space irradiation on the earth’s surface, ground–based experiments can be designed to simulate space environment using sources representing components of the relevant irradiation environment, and it is of great significance to study the physical and chemical properties of graphene under simulated space conditions.

In this paper, we investigated in detail the physical and chemical properties as well as structure of graphene before and after simulated space irradiation including AO, UV and EL to clarify whether graphene adapts to space environment. Results demonstrate that graphene possesses strong anti–irradiation capacity. In view of these properties, our attention has been focused on exploring graphene as lubricant additive for improving anti–irradiation capacity, friction reduction and antiwear abilities of lubricant. Therefore, the physicochemical and tribological properties of MACs with 0.1 wt% multilayer graphene were systematically studied under simulated space environment which is composed of HV, HT (170 °C) and LT (−100 °C), AO, UV and EL irradiation. In addition, the friction mechanism of graphene is also explored through scanning electron microscopy (SEM) and transmission electron microscopy (TEM).

## Results

### Analysis of graphene sheets and lubricant before and after irradiation

**Figure 1:**
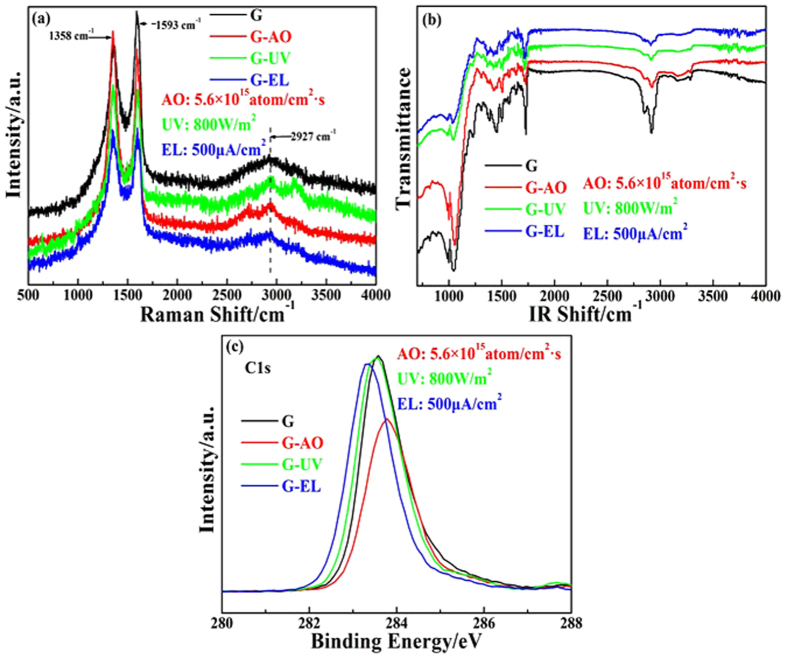
Raman spectra (**a**), Fourier transform infrared analysis spectra (**b**) and XPS spectra of C element of graphene sheets (**c**) before and after irradiation (Black, no irradiation; Red, AO; Green, UV; and Blue, EL).

Raman spectroscopy has historically played an important role in probing structural and electronic characteristics of carbon based materials. The G band (~1580 cm^−1^, in–plane vibration of sp^2^ carbon atoms), 2D band (~2670 cm^−1^, two–phonon double–resonance Raman scattering process) and D band (introduction of defects) give information about clustering of the sp^2^ phase, the presence of sp^2^–sp^3^ hybridization and impurities, and so forth^24^,^25^. The effect of irradiation on highly oriented graphene samples could be explored using Raman spectroscopy due to the specific response to the change in carbon hybridization state and the introduction of defects or foreign impurities^26^. Figure 1a gives the Raman spectra of graphene sheets before and after irradiation. For pressed graphene sheets, the D band proves the existence of some defects on the graphene basal plane and the 2D band shows the weak multiple peaks due to the influence of the decrease of interlayer spacing and the splitting of the electronic band structure of the multilayer graphene. Compared with the Raman spectroscopy of graphene samples without irradiation, the irradiation induces a significant number of defects in graphene layers which was obtained from the increase of the D band intensity at 1358 cm^−1^, the blueshift of 2D band from 2670 to 2927 cm^−1^ due to foreign atoms, electron and hole doping^27^. The energetic atomic oxygen (5 eV) with 5.6 × 10^15^ atoms/cm^2^s could react with a small number of carbon atoms activated by bombardment of high–energy particles on both basal planes and edges of graphene, which results in the modification of the flat geometric structure of graphene due to the change of the bond lengths and angles^28^. UV irradiation induced a mild structural changes of graphene because an increase of the intensity ratio of I_D_/I_G_ indicates the increased defects. Prolonged EL irradiation could lead to the appearance of vacancy– and interstitial–type defects, even the formation of carbon atomic chains^29^. Raman spectra of graphene sheets demonstrated that the stimulated space irradiation introduced some defects, whereas the structure and characteristics of irradiated graphene on average are consistent with that without irradiation, which suggests that as–prepared multilayer graphene sheets can withstand space environment.

Figure 1b shows the FTIR spectra of the samples before and after irradiation. The peak positions of the samples are consistent before and after irradiation. High–energy irradiation in vacuum could remove most of functional groups like epoxide, hydroxyl functional groups on the graphene sheets, but it may also introduce some atomic defects such as vacancies and adatoms in graphene layers that cannot be detected by FTIR spectra^30^. According to the FTIR spectra, no new peaks are detected in the samples after irradiation.

XPS is a powerful tool for characterizing the chemical state of the elements in the samples. We investigated the XPS spectrum of C element to further explore the effect of irradiation on the graphene sheet. Figure 1c gives the XPS spectra of C 1s, the strong symmetrical C 1s peaks before and after irradiation appear at the binding energy range from 283.3 to 283.8 eV, which was assigned to pure carbon of graphene^31^. XPS analysis found that high–energy particles irradiation in vacuum like EL did not lead to the formation of any new bond and only resulted in some atomic–scale defects in graphene layers, but strong oxidizing AO could react with C atoms and generate carbon–oxygen compounds like CO_2_ which was eliminated by the vacuum system.

Combined with the literature reports and the experimental results before and after irradiation, we conclude that the most important effects of high–energy particles irradiation on graphene layer are mediated through the displacement (knock–on effect) and excitation of atoms (excitation effect)^32^,^33^,^34^. The C atoms in graphene were displaced by knock–on collisions of energetic particles, which leads to the appearance of vacancies and interstitial atoms. With the increase of particles energy, their impacts could give rise to the diffusion of atoms which has the same effects as thermal diffusion. When high energy electrons bombarded the graphene sheets, the kinetic energy and charge of incident electrons could excite and ionize the C atoms, bringing them into an extremely unstable state so that recombination becomes necessary. The vacancies and interstitial atoms tend to recombine or aggregate into clusters to reduce free energy, and these excited atoms could self-assemble into pentagons or heptagons. For graphene, the excitation effect is of secondary significance since electronic excitation is rapidly weakened by conduction electrons, so knock–on effect governs the behavior of graphene and its derivatives under irradiation conditions.

**Figure 2:**
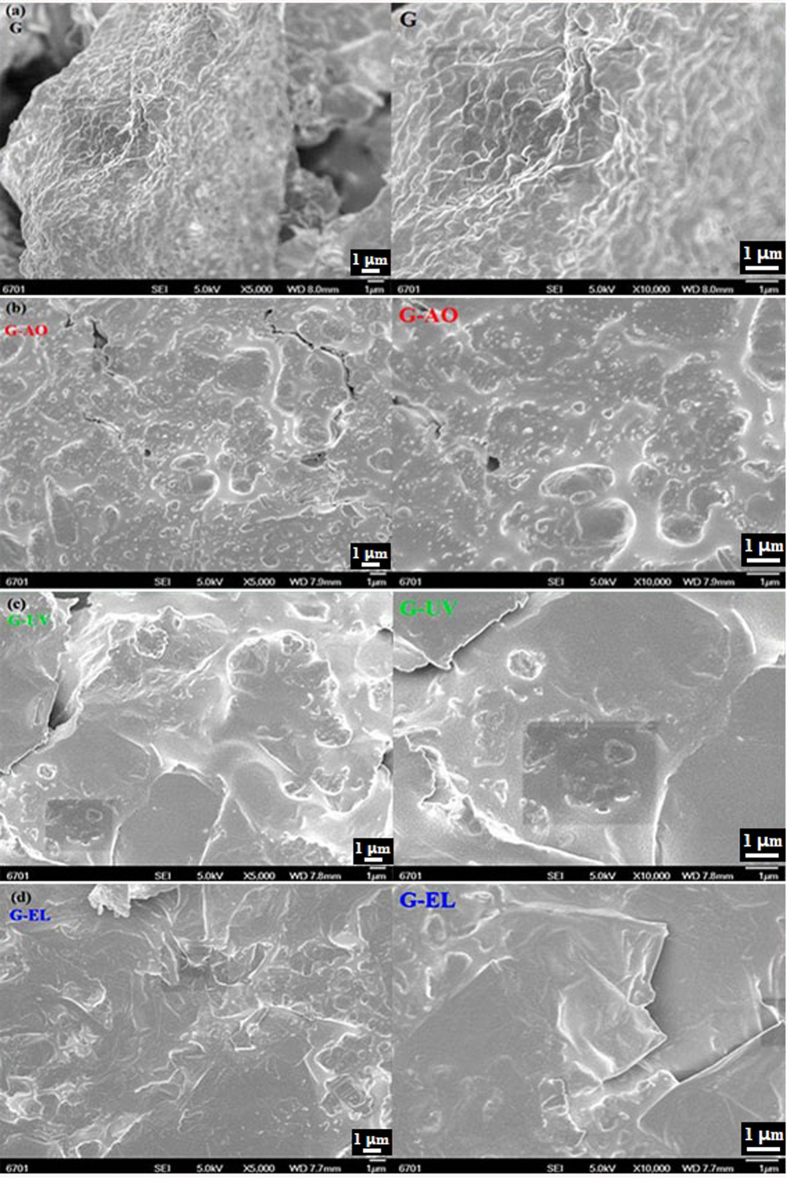
SEM micrographs of graphene sheets before and after irradiation with low/high magnification ( ×5000 and ×10000).

To visualize the morphology of graphene sheets before and after irradiation, SEM images are shown in Fig. 2. Observing these SEM images before and after irradiation, the original graphene sheets show a dense stacked layers structure, whereas the irradiated graphene sheets present relatively smooth sheet structure with some small irregular particles and suppressed graphene ripples. A large number of different sized particles, agglomerates and fragments on both basal planes and edges of the graphene sheets were observed after high–energy particles irradiation because the displaced C atoms could self-assemble into relatively stable structures or clusters between the basal planes, especially for the graphene sheets subjected to AO and irradiation^35^. The samples with UV irradiation give the most smooth surface with large sheets, which is attributed to modification of graphene by UV irradiation, for example, UV irradiation removed some oxygen-containing groups.

**Figure 3:**
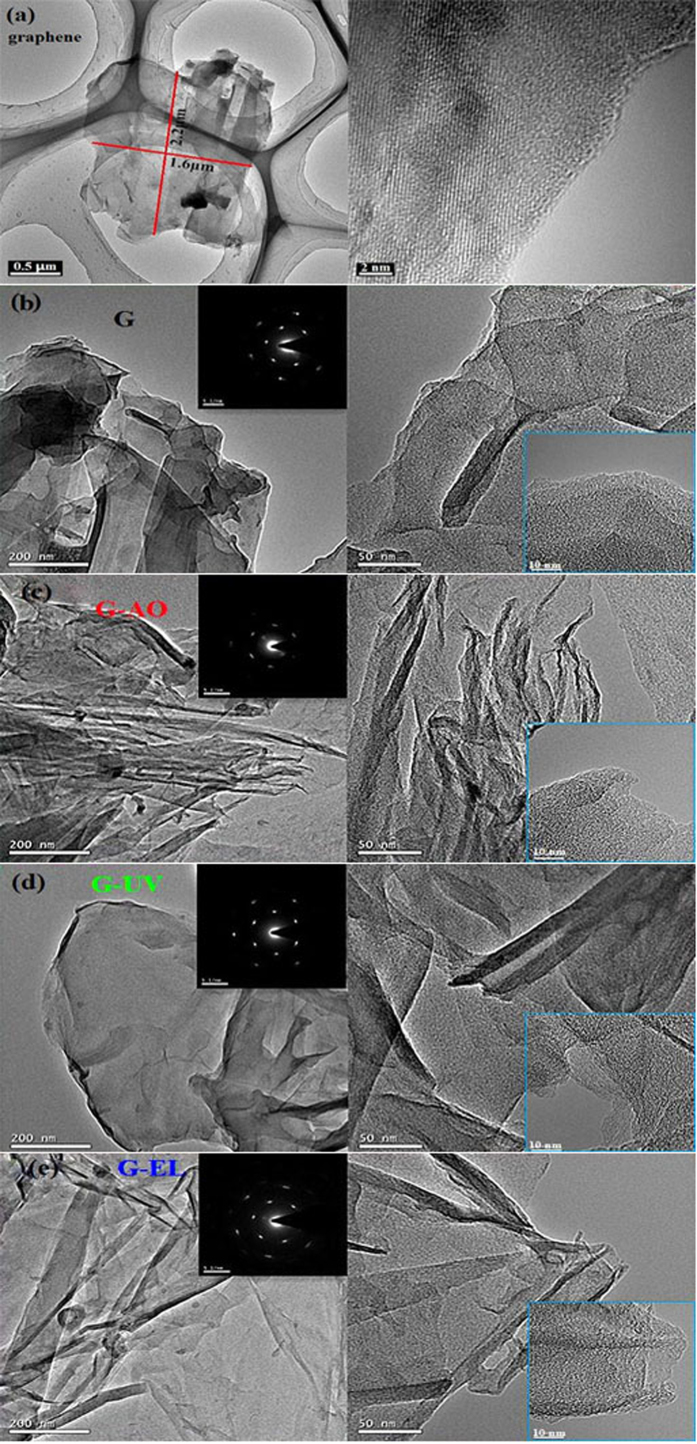
TEM micrographs of the original graphene (**a**) with its size and shape as well as HRTEM image, low and high magnification images with their selected-area electron diffraction pattern and HRTEM of the graphene sheet without irradiation (**b**), AO (**c**), UV (**d**) and EL (**e**).

TEM was employed to explore the morphology and structure of the graphene before and after irradiation. The size and shape of the original graphene is shown in Fig. 3a, it presents a clean and transparent graphene, the high–resolution micrograph indicates that the graphene is multilayer. Observing the TEM micrographs of the graphene sheets before and after irradiation, the graphene sheet (G) without irradiation (Fig. 3b) presents a relatively clean and stacked graphene layers with larger flakes. The irradiated graphene samples appear some wrinkles and curvature on the edges of graphene sheets. The bending of graphene is attributed to the introduction of defects including pentagons or heptagons, these defects can indeed be formed under high–energy irradiation because the excited C atoms via knock–on effects and excitations acquire enough energy that they can self-assemble in various structures to minimize the energy^36^,^37^. According to single layer graphene’s six–fold symmetry, its electron diffraction pattern is hexagonal^38^,^39^, and the selected area’s electron diffraction patterns of the graphene samples after irradiation are consistent with those without irradiation, which indicates that the graphene after irradiation still keeps a largely intact structure. High energy irradiation leads to the wave–like undulations of graphene planes with curvature and transformation, but no visible damage to graphene layers. Therefore, graphene can withstand the space stimulated radiation environment and can be used as lubricant additive in space.

**Figure 4:**
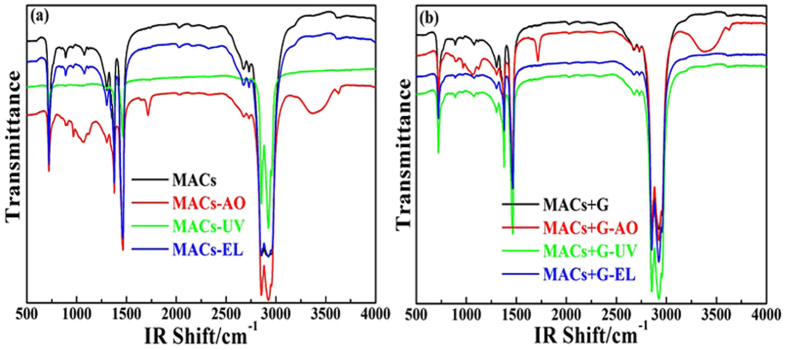
FTIR of the lubricants before and after irradiation including MACs (**a**) and MACs+G (**b**) (Black, no irradiation; Red, AO; Green, UV; and Blue, EL).

To investigate the effects of irradiation on the lubricants (MACs and MACs+G), the FTIR spectra of the lubricants before and after irradiation are shown in Fig. 4a,b. All the spectra of the lubricants are quite similar and no new peaks are detected after irradiation in vacuum except the lubricant irradiated in the presence of AO. The FTIR spectra of the lubricants after AO irradiation show some oxygen–containing functional groups, the absorption peaks at both 1070 and 1714 cm^−1^ are assigned to –C–O–C– and –CHO, a wide peak at 3380 cm^−1^ and a weak peak at 3626 cm^−1^ are due to the appearance of hydroxyl groups^40^. In addition, we notice that the peak intensity of these functional groups of the lubricants after irradiation was strengthened due to the irradiation excitation effect. The results illustrate that high energy AO caused the most serious damage to the organic lubricants via oxidation, whereas graphene could absorb the energy of irradiation and improve the lubricants anti–irradiation performance.

### Analysis of tribological properties

**Figure 5:**
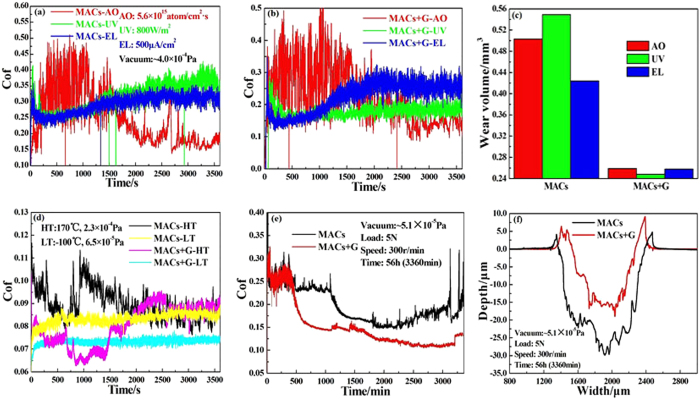
Friction curves (**a,b**) of the lubricants (MACs and MACs+G) before and after irradiation, the corresponding wear volume (**c**) (Red, AO; Green, UV; and Blue, EL), friction curves (**d**) under low and high temperature (–100 °C and 170 °C) as well as friction curves (**e**) and 2D morphology (**f**) of the worn tracks with long duration (56 h and more than one millions sliding passes).

The tribological properties of the lubricants after irradiation were systematically investigated under high vacuum condition. Figure. 5a,b depicts the friction curves of the lubricants (MACs and MACs+G) under 4 × 10^−4^ Pa condition, we could observe that the friction curves of MACs+G were much more stable, and the average friction coefficient (Cof) was always lower (~0.2) than that of MACs (~0.3) before and after irradiation. Figure 5c gives the corresponding wear volume, the wear volume of the steel disc lubricated by MACs+G (~0.26 mm^3^) is much lower than that of the disc lubricated by MACs (~0.5 mm^3^). Figure 5d shows the friction curves of the lubricants under high temperature (HT: 170 °C) with high vacuum (LT: 2.3 × 10^−4^ Pa) and low temperature (−100 °C) with higher vacuum (6.5 × 10^−5^ Pa). The lubricants showed very smooth friction curves under –100 °C and 6.5 × 10^–5^ Pa condition, and the average Cof of MACs+G was lower and more stable than that of MACs. Graphene can improve to some extent the tribological behavior of MACs under 170 °C and 2.3 × 10^−4^ Pa. The friction results in high temperature and cryogenic conditions were different because the viscosity of the lubricant largely affected the tribological performance of the systems, which mainly depends on the film thickness and fluidity of the lubricant. When friction tests were conducted in the mild test conditions or running-in stage, the good tribological performance depended on a thin oil film between the tribological interface^41^. In contrast, when the oil film reached a limiting thickness and even was damaged with increases of temperature or applied load, the anti-wear and friction-reducing behaviors in turn were dependent on graphene. Figure 5e,f show the friction curves and 2D morphology of worn tracts for long friction under high vacuum (56 h and more than one million sliding passes). With the continuous increase of sliding passes, the friction curve of MACs+G shows lower and more stable Cof due to the formation of the easily shearing graphene-rich tribo-film between the sliding surfaces, which prevented the direct contact of sliding surfaces and provided low resistance to shearing^42^. The role of MACs in improving the tribological properties is attributed to the formation and renewal of oil film on the sliding surfaces. With the continuous consumption of MACs in the process of friction, it affected the renewal of oil film and led to an unstable Cof (as shown in Fig. 5e). Figure 5f presents 2D morphology of the worn tracts lubricated by MACs and MACs+G with long durations, MACs+G possesses better wear resistance than MACs which is due to the presence of multilayer graphene. So MACs+G provides better friction reducing and antiwear performance when it is used as lubricant for steel/steel contact under simulated space environment.

**Figure 6:**
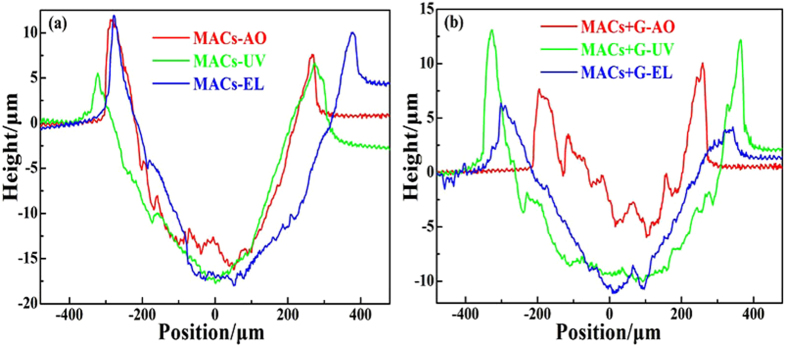
2D images of the worn tracks lubricated by MACs (**a**) and MACs+G (**b**) after irradiation under high vacuum.

In order to obtain the direct evidence on antiwear ability of MACs+G before and after irradiation, Fig. 6 displays an even closer look at the 2D topography of the wear tracks. The substrates lubricated by MACs+G show more shallow wear tracks than those lubricated by MACs, which illustrates that graphene enhances the load–bearing capacity and significantly improves the friction and antiwear behaviors.

**Figure 7:**
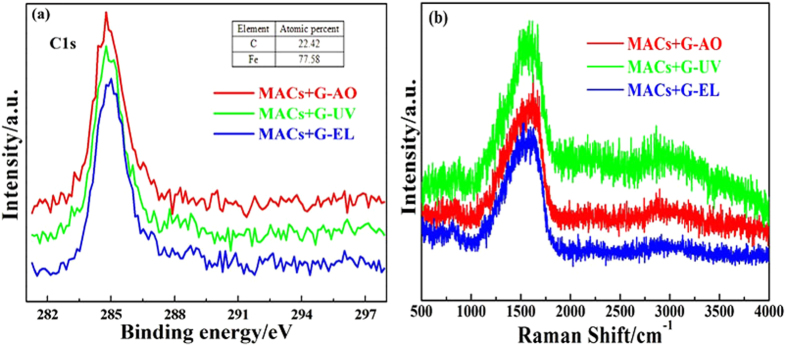
XPS spectra of C element (**a**) and Raman spectra (**b**) of the worn tracks lubricated by MACs+G with AO, UV and EL irradiation.

To further confirm the friction reducing and antiwear mechanism of MACs+G under simulated space environments, the wear tracks lubricated by MACs+G were investigated with XPS and Raman spectra and the corresponding worn particles were also analyzed with TEM. XPS and Raman spectra of wear tracks identify the components of the protective film and provide more direct evidence for the friction mechanism. As shown in Fig. 7a, the strong C1s peaks at about 284.8 eV are attributed to carbon element^43^. Observing Fig. 7b, Raman spectra further confirm the presence of graphene on the wear tracks with the relatively strong G band at ~1579 cm^−1^ and weak 2D band at~2882 cm^−1^. The disappearance of D band and broadening of G band indicate that the graphene have tended to graphitization during friction process. XPS and Raman spectra of the wear tracks demonstrate that this tribological behavior is attributed to the easily shearing graphene–rich tribo–film on the rubbing surface which prevents the rubbing surfaces from coming into direct contact, enhances the load–bearing capacity, improves the friction and wear resistance.

**Figure 8:**
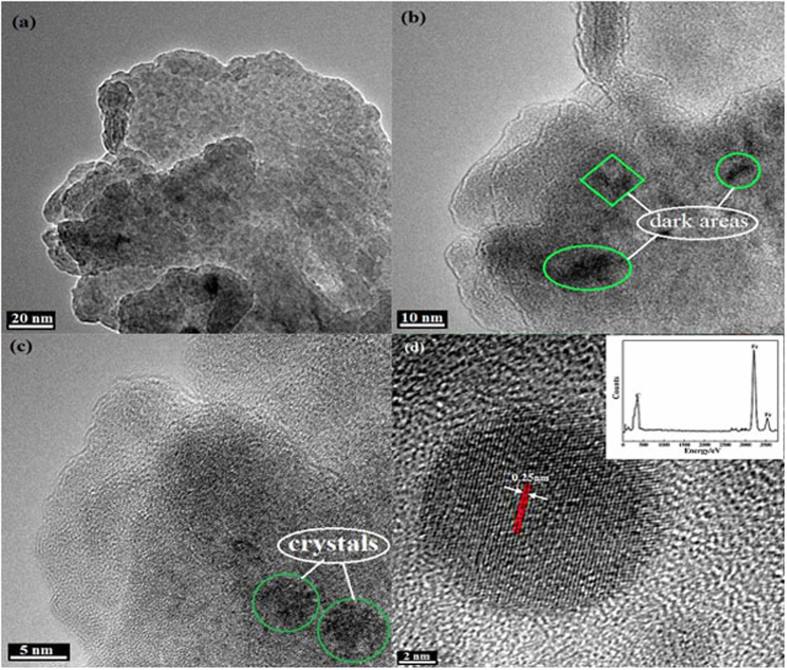
HRTEM micrographs of the worn particles with EDS elemental counts image.

TEM micrographs with EDS of the worn particles are shown in Fig. 8. As shown in Fig. 8a,b, the laminated structures of graphene sheets were clearly obtained, whereas the friction resulted in the significant increase of their thickness and the appearance of some dark areas on the graphene basal plane. To obtain additional insight into the worn particles with the dark areas, high–resolution micrographs are displayed in Fig. 8c,d, we can further confirm that the dark areas are due to the existence of the crystal structure. According to the basal plane distance of the crystal structure and EDS of the worn particles, these crystals are identified as Fe nanocrystals^44^,^45^. Results suggest that the sandwich sheets were formed through the Fe nanocrystals absorbed on the graphene basal plane and interlayer during friction process which can further enhance the load–carrying ability and improve the friction reducing and antiwear abilities.

## Discussion

**Figure 9:**
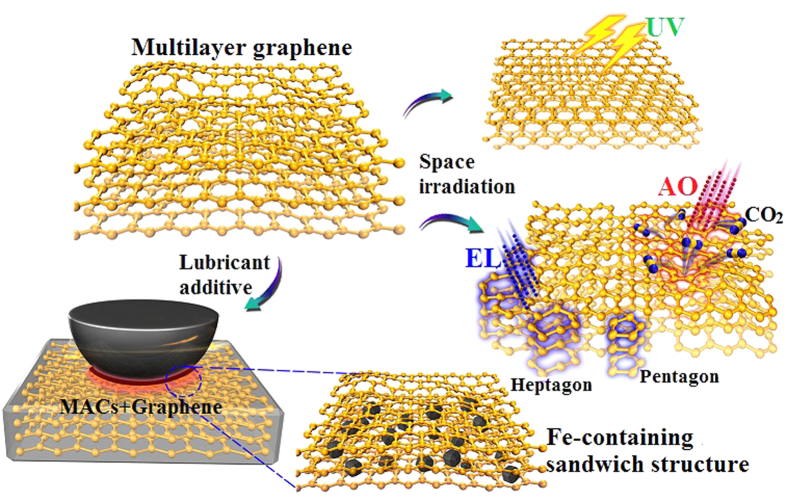
Schematic illustration about the effect of irradiation on the graphene sheets and the friction mechanism of graphene as lubricant additive.

Space environment is one of the greatest challenges for the potential applications of lubricants and lubricant additives. The effects of irradiation on graphene are systematically evaluated under simulated space irradiation environment (AO, UV and EL). Combined with these experimental results and analysis before and after irradiation as well as the literature reports^29^,^32^,^33^,^35^, the most important effects of high–energy particles irradiation (AO and EL) on graphene layer are to result in the curvature and transformation due to knock–on effect and excitation effect. Schematic illustration about the effect of irradiation on the graphene sheets and the friction mechanism of graphene as lubricant additive is shown in Fig. 9. When high–energy particles bombarded the graphene sheets, the kinetic energy and charge of incident particles can bring the C atoms into an excited and ionized state so that recombination of these atoms becomes necessary to achieve a stable state. The vacancies and interstitial atoms tend to recombine or aggregate into clusters to reduce free energy, and these excited atoms could self-assemble into pentagons or heptagons. These atomic scale defects including vacancies and interstitial atoms show no visible damage to graphene layers, which suggests that graphene has good adaptive ability to space environment because of its lamellar structure with unique physicochemical properties including outstanding thermal and electronic conductivity and good chemical inertness. Hence, graphene with such anti–irradiation capacity as space lubricant additive not only can relieve the damage of space irradiation on the lubricant through absorbing the energy of irradiation but also can significantly improve the tribological properties of the lubricant which is attributed to the easy shear capability and extreme mechanical strength of graphene. The formation of a graphene–containing protective film on sliding surfaces can provide the higher load-carrying ability and more effective separation of the sliding surfaces.

## Methods

### Irradiation procedure and characterization

Commercially obtained multilayer graphene (from Nanjing XFNANO Materials Tech Co. LTD (Nanjing, China)) was pressed into a thin sheet on the tinfoil with double–sided adhesive tape by 769YP–24Z tablet power press machine to conduct the space irradiation experiments. MACs was provided by Lanzhou Institute of Chemical Physics, Chinese Academy of Sciences (Lanzhou, China), its physical properties are shown in Table 1. MACs and MACs with 0.1 wt% graphene (MACs+G) were coated homogeneously on the stainless steel discs to carry out the space irradiation and friction experiments, respectively. Graphene (G), MACs and MACs+G were tested in the space irradiation environment including AO, UV and EL irradiation at Lanzhou Institute of Chemical Physics, Chinese Academy of Sciences.

In low earth orbit (LEO), radiation can be categorized as electromagnetic irradiation like infrared, visible, ultraviolet, X ray and gamma ray, and particulate irradiation including cosmic rays, trapped electrons and protons, and solar flares. The solar UV irradiation in the range of 100–400 nm accounts for 8% of the solar wavelength range (0.115 μm up to 50 μm), so the intensity of UV is only about 8% of the total solar energy (1366 W/m^2^)^46^. Ultraviolet can do the most damage to organic materials because absorption of ultraviolet can lead to crosslinking, chain scission, or random breaking of molecular bonds. High–energy particulate radiation is predominantly trapped radiation like electrons (up to several MeV) and protons (up to several hundred MeV). The effects of these types of particulate radiation on lubricants are ionization, phonon excitations and atomic displacement, which can result in crosslinking, chain scission or polymerization^47^,^48^. The major atmospheric constituent in LEO is atomic oxygen which originates from photodissociation of O_2_ in the upper atmosphere, its number density is about 8 × 10^7^ atoms/cm^3^ at 400 Km altitude, and the AO flux orbit is 10^14^ ~ 10^15^ atoms/cm^2^s^49^. The experimental parameters of simulated space irradiation are listed in Table 2. The experimental atomic oxygen flux was 5.6 × 10^15^ atoms/cm^2^s with impingement kinetic energy of 5 eV for 120 min at the sample position, the vacuum UV irradiation with energy levels of 800W/m^2^ in the range of 115–400 nm was carried out under high vacuum (10^−5^ Pa) for 120 min and the experiment of EL provided the high–intensity beam of electrons (500 μA/cm^2^) for 120 min. The basic principle of AO irradiation was described in ref. 50. Hence the stimulated space irradiation provides the higher energy and the stimulated experimental results are significant and helpful for space-based moving components in future space facilities.

Raman spectra of the graphene sheets before and after irradiation were first obtained by Renishaw inVia Raman microscope with 532 nm laser excitation to investigate the influence of AO, UV and EL irradiation. Fourier transform infrared (FTIR) spectra were recorded in the wavenumber range of 4000–500 cm^−1^ with Bruker IFS 66v/s fourier transform infrared analysis. Small amounts of graphene samples (5 wt‰) with KBr were finely ground in an agate mortar with a pestle for 10 minutes, then the power was filled into a drillhole of 1 cm diameter inside a stainless steel gasket and was pressed between anvils by 769YP–24Z tablet power press machine under 12 MPa for 10 s. After that, the pressure was slowly released and the as-prepared sheet with thickness of 0.5 mm was measured by FTIR. The lubricants before and after irradiation were coated homogeneously on a KBr wafer by scraper with roughness of 0.1 μm to obtain their FTIR spectra. The chemical states of the C element of graphene before and after irradiation were analyzed by a PHI–5702 multifunctional X–ray photoelectron spectroscope (XPS) manufactured by American Institute of Physics Electronics Company using K–Alpha irradiation as the excitation source. The binding energies of the target elements were determined at a pass energy of 29.3 eV, with a resolution of about ±0.3 eV, using the binding energy of oxygen (O1s: 531.0 eV) as the reference. Graphene was characterized using high–resolution TEM (FEI Tecnai F300) with an accelerating voltage of 300 kV. Small amount of graphene before and after irradiation was dispersed in ethanol in an ultrasonic bath for 20 min and deposited onto a holey carbon TEM grid. The morphology of graphene sheets before and after irradiation was observed using field–emission scanning electron microscopy (FE–SEM, JSM–6701F). The pressed graphene sheets before and after irradiation were cut into a small piece with 3 mm ×3 mm and were attached to copper disk by conducting resin. We used an accelerating voltage of 5.0 kV with low/high magnification (×5000 and ×10000).

### Tribological properties under simulated space environment

The friction tests were conducted on an in–house rotational ball–on–disk vacuum tribometer under simulated space environment including high vacuum, high/low temperature and irradiation. The fixed upper specimens were an AISI 52100 steel balls with standard 3 mm diameter, and the lower specimens were the stainless steel disks. For high/low temperature (170 °C and –100 °C) friction tests under high vacuum, the lower specimens were replaced by the steel disks (AISI 52100 steel, Φ24 × 7.9 mm with hardness of about 620 HV). Before the friction tests, the lubricants were coated homogeneously on the steel disks. All the tests were conducted at rotational radius of 7.5 mm, sliding speed of 300 r/min, applied load of 5 N and duration of 60 min under high vacuum condition. The friction coefficients were monitored continuously as a function of time. Friction test under the same conditions was repeated three times in order to ensure repeatability. The wear volume of the lower specimens and the morphology of the wear tracks were measured using a MicroXAM–3D contact surface mapping microscope profilometer. The wear tracks lubricated by MACs+G with AO irradiation were also investigated with XPS and Raman spectroscopy. The lower specimen is cleaned ultrasonically several times in baths of acetone and is dried with pure nitrogen for surface analysis. In addition, after friction tests, some worn particles were picked up from the steel disk lubricated by MACs+G with AO irradiation, they were repeatedly centrifuged with the speed of 3000 rpm for 5 min using ethanol, and were rinsed in trichloromethane and ethanol to eliminate all the residual MACs. In the end, the worn particles were investigated by TEM to further confirm the friction mechanism of graphene.

## Additional Information

**How to cite this article**: Fan, X. and Wang, L. Graphene with outstanding anti-irradiation capacity as multialkylated cyclopentanes additive toward space application. *Sci. Rep*. **5**, 12734; doi: 10.1038/srep12734 (2015).

## Figures and Tables

**Table 1:** Physical properties of multialkylated cyclopentanes (MACs).

lubricant	average molecular weight	kinematic viscosity(mm^2^/s)		viscosity index	vapor pressure (Pa) at 20 °C
MACs	630	40 °C	100 °C	146	5.6 × 10^−6^
		55.8	9.2		

**Table 2:** Experimental parameters of space irradiation.

irradiation	parameters	friction condition
AO	4.6 × 10^15 ^atom/cm^2^·s, 5 eV, 2 h	5 N, 300 r/min, 60 min, 4 × 10^−4^ Pa
EL	500 μA/cm^2^ ,2 h	5 N, 300 r/min, 60 min, 4 × 10^−4^ Pa
UV	800 W/m^2^, 115 nm–400 nm, 2 h	5 N, 300 r/min, 60 min, 4 × 10^−4^ Pa
